# Effect of codon optimization and subcellular targeting on *Toxoplasma gondii *antigen SAG1 expression in tobacco leaves to use in subcutaneous and oral immunization in mice

**DOI:** 10.1186/1472-6750-10-52

**Published:** 2010-07-15

**Authors:** Melina Laguía-Becher, Valentina Martín, Mauricio Kraemer, Mariana Corigliano, María L Yacono, Alejandra Goldman, Marina Clemente

**Affiliations:** 1 IIB-INTECH, Camino de Circunvalación km 6, Provincia de Buenos Aires, Argentina; 2 Escuela de Ciencia y Tecnología, UNSAM, Av. Gral. Paz 5445, San Martin, Argentina

## Abstract

**Background:**

Codon optimization and subcellular targeting were studied with the aim to increase the expression levels of the SAG1_78-322 _antigen of *Toxoplasma gondii *in tobacco leaves. The expression of the tobacco-optimized and native versions of the *SAG1 *gene was explored by transient expression from the *Agrobacterium tumefaciens *binary expression vector, which allows targeting the recombinant protein to the endoplasmic reticulum (ER) and the apoplast. Finally, mice were subcutaneously and orally immunized with leaf extracts-SAG1 and the strategy of prime boost with rSAG1 expressed in *Escherichia coli *was used to optimize the oral immunization with leaf extracts-SAG1.

**Results:**

Leaves agroinfiltrated with an unmodified *SAG1 *gene accumulated 5- to 10-fold more than leaves agroinfiltrated with a codon-optimized *SAG1 *gene. ER localization allowed the accumulation of higher levels of native SAG1. However, no significant differences were observed between the mRNA accumulations of the different versions of SAG1. Subcutaneous immunization with leaf extracts-SAG1 (SAG1) protected mice against an oral challenge with a non-lethal cyst dose, and this effect could be associated with the secretion of significant levels of IFN-γ. The protection was increased when mice were ID boosted with rSAG1 (SAG1+boost). This group elicited a significant Th1 humoral and cellular immune response characterized by high levels of IFN-γ. In an oral immunization assay, the SAG1+boost group showed a significantly lower brain cyst burden compared to the rest of the groups.

**Conclusion:**

Transient agroinfiltration was useful for the expression of all of the recombinant proteins tested. Our results support the usefulness of endoplasmic reticulum signal peptides in enhancing the production of recombinant proteins meant for use as vaccines. The results showed that this plant-produced protein has potential for use as vaccine and provides a potential means for protecting humans and animals against toxoplasmosis.

## Background

The use of plants for the large-scale production of heterologous proteins is gradually gaining widespread acceptance and could provide a platform for the cost-effective production of proteins on an agricultural scale. In particular, it has been proposed that plant production for human and animal vaccines may significantly lower the cost of production of the raw material, especially for oral vaccination [1,2]. However, low protein yield is a significant problem limiting the commercial exploitation and the competition with other heterologous production methods [3]. In this sense, several approaches have been developed to increase protein expression in plants. In particular, techniques such as codon optimization and subcellular targeting can markedly improve the expression levels [4].

*Toxoplasma gondii *is an obligate intracellular parasite capable of infecting a variety of mammals, including humans, and birds [5]. In humans, toxoplasmosis is usually asymptomatic in healthy individuals. However, in pregnant women, congenital infection can cause severe neonatal malformations and ocular complications in the fetus; and in immunocompromised individuals, such as AIDS patients and transplant recipients, often results in fatal encephalitis [6,7]. Toxoplasmosis is also of veterinary importance, especially in sheep and pigs, where it often results in abortion, causing considerable economic losses [8,9]. In addition, the tissue cysts of *T. gondii *in meat of infected livestock are an important source of infection for humans [10,11].

Although a vaccine against human infection with *T. gondii *is not yet available, an effective vaccine preventing infection in animals used for human consumption would block the main transmission route to humans [12]. Also, in the farming industry, a vaccine against *T. gondii *may be valuable to prevent both fetal infection and reactivation.

Recently, we transiently expressed the surface antigen 1 (SAG1) and granule dense 4 (GRA4) of *Toxoplasma gondii *in tobacco leaves [13,14]. SAG1 protein is the best characterized and the primary vaccine candidate [13,15-18]. Numerous studies have demonstrated its vaccine potentialities as a recombinant protein or as a DNA vaccine [11,19-23]. In fact, a SAG1-specific CD8+ T-cell clone exhibited cytolytic activity against target cells infected with *T. gondii *[24]. In an effort to improve the expression level of SAG1 in plant leaves, in the present work, we investigated the effects that codon optimization and plant cell-compartment targeting have on SAG1 expression levels using vacuum agroinfiltration. We also evaluated the efficacy of subcutaneous or oral immunization with SAG1-expressing leaf extract.

## Results

### Synthesis of a codon-optimized *SAG1 *gene

**Figure 1:**
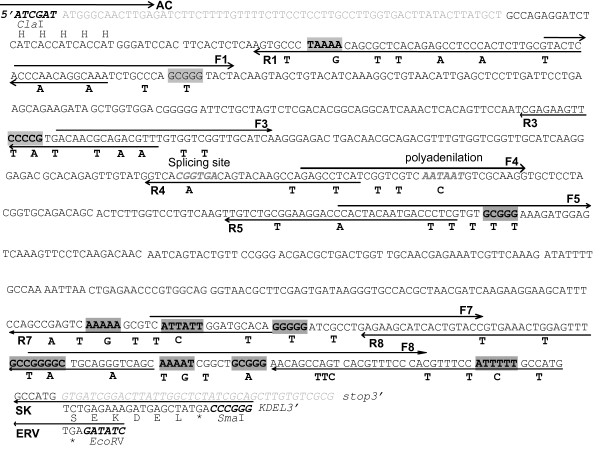
**Characteristics of the synthetic *SAG1 *gene**. Nucleotides sequence of the native SAG1gene including the *Cla*I site at the start, and *Sma*I and *EcoR*I sites beyond the stop codons. The apoplastic and endoplasmic reticulum signal sequences at 5' and 3', respectively, are indicated in gray letters. The differences between the synthetic gene used in this study and the wild-type gene are denoted below the nucleotide sequence of the latter. The His-tag coding sequence is indicated as H. Gray boxes indicate consecutive A+T or G+C nucleotides of 5 bp or more.

SAG1, previously named P30 (accession no. GQ253098), is the main surface antigen of *T. gondii *[25]. In the parasite, SAG1 is processed before it is loaded to the plasma membrane, losing the signal peptide and the C-terminal hydrophobic region, to generate the mature SAG1_77-322 _version [25]. For this reason, the open reading frame sequence encoding the native SAG1_77-322 _(nS) was used as a source of codon optimization for expression in tobacco (Table 1). A total of 57 codons were altered in the nS sequence to match the codon usage preferences of *Nicotiana tabaccum*, yielding an optimized-plant version of the SAG1 sequence named oS (Figure 1 and Table 1). The *oS *gene was synthesized using a set of overlapping oligonucleotides (Figure 1 and Table 2). The optimization strategy removed only about 20% of all the infrequently used codons. In addition, in order to avoid potential negative factors influencing protein expression, a putative splicing site, a potential plant polyadenylation signal sequence, consecutive strings of A + T or G + C nucleotides of 5 bp or more were removed (Figure 1). The final G + C content of the *oS *gene was lowered to 46% and the codon adaptation index value (CAI) was increased to 0.83 for matching with tobacco genes as compared to the 0.67 CAI value of the nS sequence. The nucleotide sequence of the *oS *gene (accession no. GU191164) was 92% homologous to the *nS *gene. However, the amino acid sequence encoded by the *oS *was identical to that of the *nS *gene to ensure retention of the antigenicity of the expressed protein.

**Table 1:** Summary of *Agrobacterium *expression constructs used during this study

Vector	Insert	clone name	Protein name	cell compartment targeted
pZPVX	SAG1 (full-length protein)	pZPVXSAG1^a^	PVXS	Cytoplasm
Pzp	SAG1 (full-length protein)	pApoSAG1^a^	AS	Apoplast
Pzp	nS (last 14 codons removed)	pAnS	AnS	Apoplast
Pzp	oS (last 14 codons removed and plant-optimized SAG1)	pAoS	AoS	Apoplast
Pzp	KnS (last 14 codons removed)	pKnS	KnS	Endoplasmic reticulum
Pzp	KoS (last 14 codons removed and plant-optimized SAG1)	pKoS	KoS	Endoplasmic reticulum

^a ^[13]

**Table 2:** *Primers *used in the PCR amplification to obtain the optimized SAG1_(77-322) _sequence.

Primer name	Length	Sequences
AC	26 bp	5' atcgatatgggcaacttgagatcttc 3'
ERV	39 bp	5' gatatcTCAcatAgcaaaGatAgaaacAtgaGAAgctgt 3'
SK	57 bp	5' cccgggtcatagctcatctttctcagacatAgcaaaGatAgaaacAtgaGAAgctgt3'
F1	36 bp	5' tactcaccAaacagAcaaatctgTccagcTggtact 3'
R1	54 bp	5' TctgttTggtgagtaAgcaagagtTggaggTtctgtAagAgctgtCttaggAca 3'
F3	30 bp	5' acaacTcaAacAtttgtTgtTggttgcatc 3'
R3	30 bp	5' aaaTgtTtgAgttgtAacTggAaacttctc 3'
F4	33 bp	5' caagcTagagcctcatcTgtTgtcaaCaatgtc 3'
R4	33 bp	5' AgatgaggctctAgcttgAactgtcacTgtgac 3'
F5	39 bp	5' ggaccAactacaatgacTctTgtTtgTggTaaagatgga 3'
R5	36 bp	5' AagAgtcattgtagtTggtccttcAgcagacaactt 3'
F7	48 bp	5' gtTatCattggatgTacaggTggatcTcctgagaagcatcactgtacT 3'
R7	39 bp	5' AcatccaatGatAacActCttAgaTtcAgctggaaatgc 3'
F8	48 bp	5' ggAgctgcaggAtcagcTaaGtcTgctgcAggaacagcTTCtcaTgtttcT 3'
R8	48 bp	5' tgaTcctgcagcTccAgcaaactcAagtttcacAgtacagtgatgctt 3'

Recognition site for the endonuclease *Cla*I, *EcoR*V and *Sma*I indicated underlined in AC, ERV and SK primers, respectively. Specific mutation sites are indicated in capital letters.

### Construction of the apoplast and endoplasmic reticulum targeting vectors

**Figure 2:**
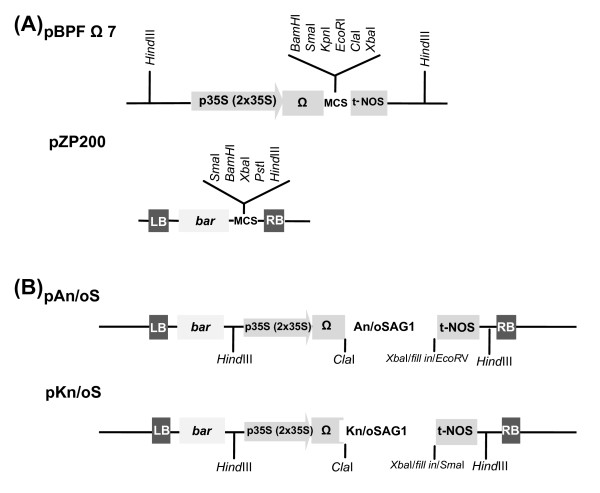
**Schematic representation of constructs used in the *Agrobacterium*-mediated transient expression systems**. (A) Schematic representation of the expression cassette of intermediate plasmid pBPFΩ7 and the binary plant vector pZP200. (B) Schematic representation of pAn/oS and pKn/oS construct. An/oSAG1: the native and plant-optimized genes (residues 77 to 322, excluding the signal peptide in the N-terminus and the 14 hydrophobic amino acids in the C-terminus) fused to the AP24 osmotin signal peptide sequence in the N-terminus for driving the mature protein to the apoplast. Kn/oSAG1: the native and plant-optimized genes (residues 77 to 322) fused to the AP24 osmotin and an endoplasmic reticulum retention signal peptide sequences (TCTGAGAAAGATGAGCTAT) in the N-terminus and the C-terminus, respectively, for accumulation of the recombinant protein in the RE. 35sCaMVx2: duplicated CaMV 35S promoter; T-nos: nopaline synthase terminator sequence; Ω: translational enhancer sequence; *bar: *gluphosinate-resistant gene; RB and LB: right and left borders of the T region, respectively; MCS: multiple cloning site.

The oS and nS sequences were cloned in a pzp200bar binary vector for plant expression (Figure 2A and Table 1). Both the oS and nS sequences were fused to the tobacco (*Nicotiana tabaccum*) AP24 osmotin signal peptide sequence at the N-terminus end to target the recombinant proteins to the apoplast, generating the AoS and AnS sequences (Figure 2B and Table 1). Combining the apoplast targeting sequence with an endoplasmic reticulum retention signal (KDEL) incorporated at the C-terminus end the recombinant proteins are sequestered in the ER of plant cells (KoS and KnS sequences, Figure 2B and Table 1).

The pzp200bar binary vector, where the AoS, AnS, KoS and KnS versions were cloned, contains the duplicate cauliflower mosaic virus 35S promoter (35SCaMV × 2) with the translational enhancer sequence (Ω) and the nopaline synthase (nos) terminator to stop transcription. Pzp200bar also contains the BASTA resistance marker (*bar *gene), which permits the selection of transformed plants (Figure 2). In addition to these constructions, the previously pZPVXSAG1 and pApoSAG1 constructs [13], where SAG1 is expressed as a full-length protein lacking the signal peptide (SAG1_77-336_: from position 77 to the end 336), were also used (Table 1).

### Transient expression of SAG1 in plants

Vacuum agroinfiltration was used to compare the expression of SAG1 constructs in plants. Recombinant *Agrobacterium *carrying these constructs and the control pzp200 were used to agroinfiltrate leaves of the *Nicotiana tabaccum cv. Xanthi transgenic line X-27-8 *[26]. This line expresses high levels of the *Tobacco etch virus *(TEV) P1/HC-Pro sequence. *Agrobacterium*-mediated transient expression declines sharply as a result of post-transcriptional gene silencing (PTGS). Certain plant viruses encode silencing suppressors that may inhibit PTGS and are used to extend and enhance *Agrobacterium*-mediated transient expression [27]. In a previous work, SAG1 was successfully expressed in this transgenic line [13].

**Figure 3:**
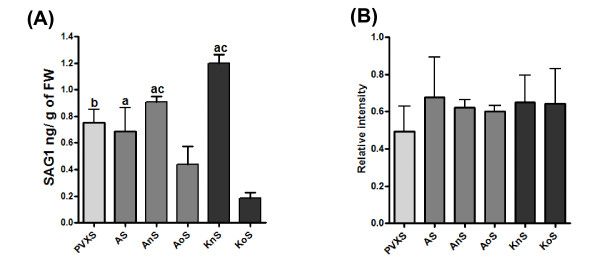
**Effect of intracellular localization and codon modification on accumulation of SAG1**. (A) SAG1 accumulation in tobacco leaves. The SAG1 band intensity expressed from *E. coli *and detected by Western blot was estimated to build a quantization calibration curve with amount standards. The leaf-expressed SAG1 band intensity detected by western blot was estimated using "Gel-Pro analyzer" and compared with the quantification calibration curve; a = p < 0.05: KnS vs. PVXS and AS, AS vs. KoS, and AnS vs. AoS; b = p < 0.01: PVXS vs. KoS; c = p < 0.001: AnS vs. KoS, and KnS vs. AoS and KoS. FW: fresh weight, M, molecular weight marker pre strained (Fermenta); (B) Relative amounts of SAG1 transcripts. SAG1 transcript levels were normalized with the actin transcripts used as an internal control using "Gel-Pro analyzer". Statistical analysis was performed by one-way analysis of variance (ANOVA) using the Newman-Keuls Multiple Comparison Test.

SAG1 protein expression was measured at 4 days post-infiltration by Western blot from three independent *Agrobacterium *infiltrations. In agreement with our previous work [13], Western blot analysis of infiltrated tobacco leaf samples demonstrated the successful expression of the 35 kDa SAG1 together with another specific band of about 19 kDa (Additional file 1A). SAG1 accumulation in tobacco leaves was estimated by Western blot analysis (Additional file 1B) using the Gel-Pro analyzer program as described in methods. Agroinflitration with the pKnS vector produced the highest level of SAG1 expression being the accumulation of the KnS protein 5- and 10-fold higher than AoS and KoS, respectively (Figure 3A and Additional file 1C). In fact, the KnS yields were ~1.3 μg per gram of fresh weight (FW), while the AoS and KoS vectors generated the lowest SAG1 production: ~0.15 μg and ~0.4 μg per gram of FW, respectively (Figure 3A). On the other hand, the pAnS and pApoSAG1 vectors expressed intermediate SAG1 levels (Figure 3A and Additional file 1C). The apoplast-localized native protein levels were ~0.7 μg per gram of FW, near a production 50% lower than that obtained by pKnS (Figure 3A). Finally, the removal of the C-terminus did not affect its accumulation, when compared with its full-length (AnS vs. AS) (Figure 3A).

In order to know whether the differences in protein expression were due to differences in mRNA expression, a semi quantitative RT-PCR was performed (Additional file 1D). SAG1 mRNA expression was measured at 3 days post-infiltration from three independent *Agrobacterium *infiltrations. SAG1 transcript levels were normalized with the actin transcripts used as an internal control. To estimate the level of SAG1 mRNA accumulation, the band intensity was quantified by the Gel-Pro program and normalized to the band intensity of actin mRNA quantified by the same program. Relative mRNA units from the three independent *Agrobacterium *infiltrations were plotted in Figure 3B. Statistical analysis from the data of the three independent agroinfiltrations altogether showed that the level of SAG1 mRNA accumulation among the infiltrated leaves with the different constructs was similar (Figure 3B).

### Immunogenicity of plant-derived SAG1

**Figure 4:**
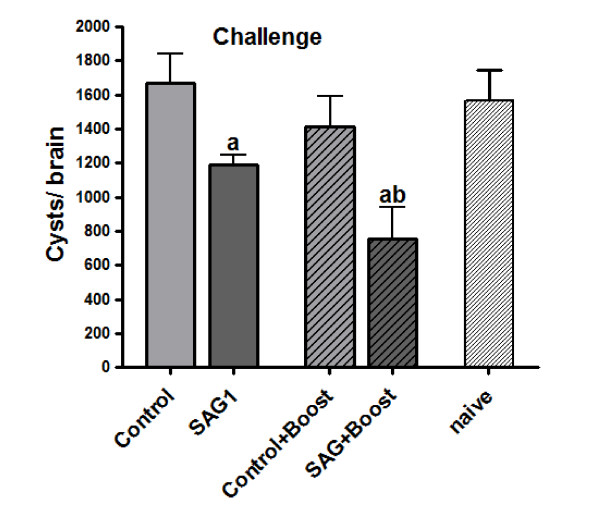
**Protection of C3H/HeN mice against *Toxoplasma *infection**. Eight- to ten-week-old mice (8/group) were immunized on days 0, 14, 28 and 42 by subcutaneous injection. Two weeks after the last boost, mice were challenged by gavages with 20 cysts of the Me49 strain (LD50). Thirty days after the challenge, the number of brain cysts in mice was determined. Each bar represents the group mean ± S.E.M.a = p < 0.05: SAG vs. Control and naïve, and SAG1+Boost vs. Control+Boost; b = p < 0.01: SAG1+Boost vs. Control and SAG1+Boost vs. naïve. Control: mice vaccinated with pzp200-infiltrated leaf extracts, SAG1: pKnS-infiltrated leaf extracts, Control+Boost: pzp200-infiltrated leaf extracts + rSAG1 prime boost, SAG1+Boost: mice vaccine with pKnS-infiltrated leaf extracts plus rSAG1 prime boost. The results represent one of two similar experiments. Statistical analysis was performed by one-way analysis of variance (ANOVA) using the Bonferroni's Multiple Comparison Test.

As a first approach to study the immunogenicity of the plant-derived SAG1, C3H/HeN mice were subcutaneously immunized with leaf extracts expressing KnS protein crude extract combined with incomplete Freund adjuvant (IFA) (SAG1 group). Mice inoculated with leaves infiltrated with the empty pzp200 plasmid plus IFA (Control group) were used as controls. A naïve group was also included as an additional control. To analyze the immunoprotective value, two weeks after the last immunization, mice were orally challenged with a non-lethal dose of ME49 cysts and one month later the brain parasite burden was measured. The SAG1 group showed a significant reduction in the brain cyst burden (30%) compared to control and naïve groups while these two last groups presented a similar brain parasite load (Figure 4). In order to improve the immune protection obtained in SAG1 immunized animals, a prime-boost immunization protocol was assayed. Both the SAG1 and Control groups were boosted intradermally (ID) with rSAG1 expressed in *E. coli *plus IFA (SAG1+boost and Control+boost, respectively). This protocol resulted in a significant reduction of 46% in the brain cyst burden compared to the Control+boost group and of 54% compared to naïve mice (Figure 4). No significant differences were detected between the SAG1 and SAG1+boost groups or between the control and Control+boost groups.

**Figure 5:**
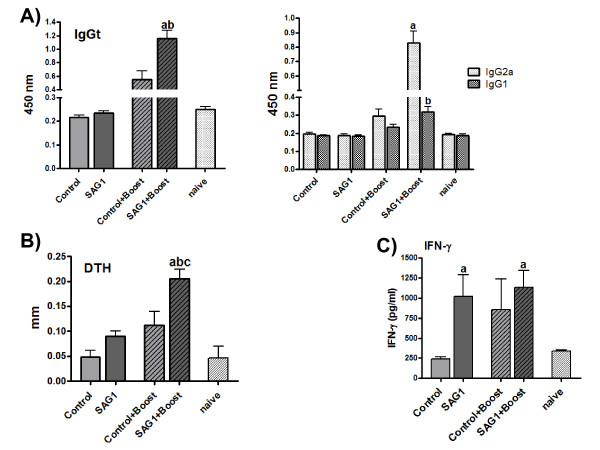
**Humoral and cellular response in C3H/HeN vaccinated mice**. (A) Determination of specific anti-rSAG1 humoral response in C3H/HeN mice. Serum IgG profile in immunized mice determined by ELISA. IgGt: a = p < 0.01: SAG1+Boost vs. Control+Boost; b = p < 0.001: SAG1+Boost vs. Control, SAG1 and naïve; IgG2a: a = p < 0.001 SAG1+Boost vs. Control, SAG1, Control+Boost and naïve; IgG1: b = p < 0.01 SAG1+Boost vs. Control, SAG1 and naïve. Values for each serum sample were determined in duplicate. (B) Delayed-type hypersensitivity (DTH) to *Toxoplasma gondii *48 h post-intradermal injection in mice. a = p < 0.05: SAG1+Boost vs. Control+Boost; b = p < 0.01: SAG1+Boost vs. SAG1; c = p < 0.001: SAG1+Boost vs. Control and naïve. (C) Cytokine production by splenocytes from vaccinated mice. Cells were harvested two weeks after the last immunization and cultured in the presence of rSAG1 (10μg/ml). Supernatants were collected 72 h later and assessed for the production of IFN-γ by capture ELISA. a = p < 0.05: SAG1 and SAG1+Boost vs. Control and naïve. Control: mice vaccinated with pzp200-infiltrated leaf extracts, SAG1: pKnS-infiltrated leaf extracts, Control+Boost: pzp200-infiltrated leaf extracts + rSAG1 prime boost, SAG1+Boost: mice vaccine with pKnS-infiltrated leaf extracts plus rSAG1 prime boost. Results are expressed as the means value ± S.E.M and represent one of two similar experiments. Statistical analysis was performed by one-way analysis of variance (ANOVA) using the Bonferroni's Multiple Comparison Test.

Two weeks after the immunization schedule was completed, blood samples were collected and analyzed by ELISA for specific anti-SAG1 responses. Also, cellular responses were studied *in vivo *by a DTH reaction and *in vitro *by the characterization of the cytokine profile secreted by splenocytes (Figure 5). Although SAG1-immunized mice showed neither a significant IgG production compared to the control and naïve groups (Figure 5A) nor a positive DTH reaction (Figure 5B), significant levels of IFN-γ were detected in supernatants of spleen cells after *in vitro *stimulation with rSAG1 (Figure 5C).

On the other hand, SAG1+boost-immunized mice elicited significant Th1 polarized humoral and cellular responses (Figure 5). Significant levels of specific IgG were detected compared to all the experimental groups, with predominant secretion of IgG2a over the IgG1 isotype (Figure 5A). SAG1+boost immunized animals also showed an antigen-specific cell-mediated immunity as measured by a positive DTH reaction that resulted significantly different compared to all the other groups (Figure 5B). Upon antigen-specific *in vitro *stimulation, the levels of IFN-γ secretion were higher than in naïve and control mice (Figure 5C). The inclusion of an ID boost with rSAG-1 in control animals (Control+boost) induced an increase in specific IgG levels and DTH reactivity, but in contrast to the SAG1+boost group, these differences did not significantly differ from the control and naïve groups (Figure 5A, B). Regarding IFN-γ secretion, the Control+boost group presented increased levels of this cytokine, which resulted similar to that observed in the SAG1 and SAG1+boost groups, but not significantly different from control and naïve mice (Figure 5C).

**Figure 6:**
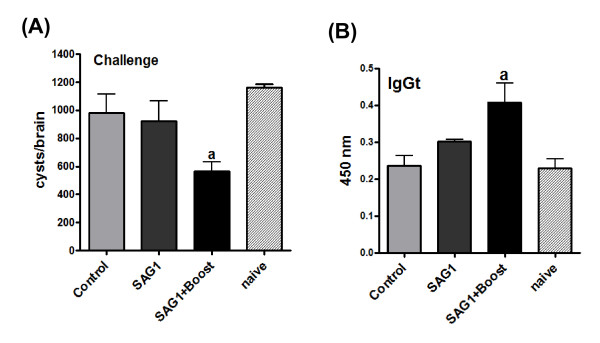
**Humoral response in orally immunized C57BL/6(H-2d) mice and protection assay after a challenge with *T. gondii *cysts**. (A) Cyst number per brain. a = p < 0.05: SAG1+Boost vs. SAG1, Control and naïve. (B) Determination of specific anti-rSAG1 humoral response in C57BL/6 (H-2d) mice. Specific IgGt titers in sera from vaccinated mice were determined by ELISA. a = p < 0.001: SAG1+Boost vs. SAG1, Control and naïve. Control: mice vaccinated with pzp200-infiltrated leaf extracts, SAG1: pKnS-infiltrated leaf extracts, SAG1+Boost: mice vaccinated with pKnS-infiltrated leaf extracts plus rSAG1 prime boost. Statistical analysis was performed by one-way analysis of variance (ANOVA) using the Bonferroni's Multiple Comparison Test. Results are expressed as the means value ± S.E.M and represent one of two similar experiments. Values for each serum sample were determined in duplicate.

Considering that *T. gondii *infects the host through the gut mucosa, the feasibility of inducing immunogenicity at this site by the use of transgenic plants in oral vaccination strategies is an interesting tool. Therefore, an oral immunization protocol was included in this study using C57BL/6 (H-2d) mice. Three experimental groups were used: mice orally immunized with SAG1-expressing leaf extract (SAG1), mice orally immunized with pzp200-infiltrated leaf extracts (Control), and a third group of mice orally immunized with SAG1-expressing leaf extracts plus an intradermal boost with rSAG1 expressed in *E. coli *(SAG1+boost). To evaluate the protection against *T. gondii *infection, two weeks after the vaccination schedule was completed, immunized mice were orally challenged and the number of *T. gondii *brain cysts was analyzed one month later. No protection was observed in SAG1-vaccinated mice compared to the control and naïve groups (Figure 6A). In contrast, the SAG1+boost group presented a significant reduction in the parasite load (50%) when compared to both the SAG1 and control groups (Figure 6A). The number of brain cysts in the control (Figure 6A and Additional file 2) or PBS+boost (Additional file 2) groups was not different from that obtained in naïve mice. In the SAG1+boost group, a significant increase in the SAG1-specific IgGt was observed when compared to the SAG1 and control groups (Figure 6B). However, we could not detect significantly increased levels in any of the IgG isotypes or a DTH positive response in any of the experimental groups (data not shown).

## Discussion

Our group has previously investigated the suitability of a vacuum infiltration system to express SAG1 in tobacco leaves [13]. In that study we tested the performance of three different constructs. Two of them were based on the PVX amplicon carrying the *SAG1 *gene and the other corresponded to pApoSAG1 [13]. Although the SAG1 accumulation levels were similar in the PVX amplicon (PVXS) and pApoSAG1 (AS), the use of this amplicon in transgenic plants is more difficult due to the induction of post-transcriptional gene silencing [26,28]. For this reason, in order to improve the nuclear expression of SAG1 in plants, in this work, other binary vectors containing codon-optimized *SAG1 *genes and plant cell compartment-targeting signal sequences were investigated using transient expression in *Nicotiana *by vacuum agroinfiltration. In addition, the new constructs deleted the DNA sequence encoding the highly hydrophobic 14 C-terminal residues that are processed in the native SAG1 protein [29].

In general, rare codons, AU-rich destabilizing sequences and putative polyadenylation and splicing signals may contribute to rapid mRNA decay, thus limiting the expression of foreign genes in plants. The CAI value is considered a measure of the expression levels of a given gene in different organisms. It is believed that increasing the CAI of a foreign gene results in a high expression level of their proteins. However, the increase in the CAI value brings about an increase in the A+T content, which could decrease mRNA stability and reduce the protein expression level [30-34]. Recently, Sou et al. [35] have shown that constructs carrying optimized sequences, where the A+T content is higher than 50%, present a protein expression level significantly lower than the native protein, although the mRNA level by RT-PCR is similar in all the constructs. The human papillomavirus type 16 (HPV-16) L1 gene re-synthesized to reflect human codon usage expresses better than the native gene, which expresses better than a plant-optimized gene [36]. Thus, the highest expression of human L1 protein is correlated with a lowest A+T content, resulting in a better transcription and RNA processing. Finally, in the codon-optimized PyMSP4/5 antigen from *Plasmodium*, the A+T content is reduced from 67% to 53% and the AT-rich regions are disrupted [37,38]. The constructs that express a reduced A+T gene version are more efficient in antigen production than native versions, supporting that the increase in A+T content may decrease mRNA stability and reduce the protein expression level [37,38]. In our study, codon optimization allowed increasing the CAI value and A+T content for matching with tobacco genes. In the *AoS *and *KoS *DNA sequences, the A+T content (54%) was higher than that in the *AS*, *AnS *and *KnS *(48%) genes and the CAI value was also increased to 0.83. Although this optimization of the *SAG1 *gene did not significantly modify the mRNA levels, the plant-optimized SAG1 expression was significantly lower than KnS, AnS and AS versions. Based on that, we could infer that plant codon optimization negatively affected SAG1 expression, being the yield of optimized protein extremely low without apparently affecting the transcript accumulation. However, further studies should be carried out to shed light on this point.

According to the SAG1 accumulation analysis by western blot, we can group the expression systems in three categories; higher expression (KnS), intermediate expression (PVXS, AS, AnS) and lower expression (KoS, AoS). These results indicate that ER retention of SAG1 is highly positive and that the low expression level of the KoS version seems to be restricted to the translational step. Proteolysis is more likely to occur in the apoplast rather than in the ER [3,4,39,40]. Thus, retention of the protein in the ER can be used to minimize foreign protein degradation since the ER provides a relatively protective environment with molecular chaperones and stabilizing agents, which results in an increase of protein stability and an enhanced level of protein accumulation. On the other hand, SAG1 yield was lower in the cytoplasm using PVX amplicon than in the ER using nuclear expression systems. Besides, the use of PVX amplicon induces gene silencing in transgenic plants that considerably affects the transgene expression, being the heterologous protein sometimes undetectable. For this reason, to produce recombinant proteins in transgenic plants, they must be co-expressed with proteins able to suppress this mechanism. However, this co-expression makes this procedure rather difficult and little feasible. Overall, our data indicate that the best choice for plant expression is the KnS system.

This study also focused on improving the immunization strategy with the leaves containing the SAG1 protein and was set up as a proof of concept that plant-produced *T. gondii *proteins could possibly be further developed as candidates for vaccine approaches. Previously, we had demonstrated that C3H/HeN mice subcutaneously vaccinated with SAG1 expressed in tobacco leaves showed significant lower brain cyst burdens compared to the control group [13]. In that work only the humoral response was analyzed showing that a weak Th1 polarized response was elicited. To further evaluate the immunogenic value of plant-produced SAG1 antigen, in the present study we characterized the immune responses elicited by the extract from leaves infiltrated with the pKnS using different immunization protocols. As in our previous study, significant partial protection was obtained in SAG1 immunized mice compared to the controls. Although we could not detect specific IgGs antibodies, SAG1 immunization elicited a Th1 cellular response characterized by significant IFN-γ production in splenocyte supernatants. A vaccine candidate against toxoplasmosis has to primarily elicit a Th1 humoral response and especially increase IFN-γ levels, since this cytokine is one of the main mediators in host resistance to *T. gondii *[41]. In our case, no specific-humoral response was detected and this could be due to the fact that SAG1 protein possesses conformational B-cell epitopes [22,42]. Therefore, as the SAG1 present in the leaf extracts stimulated only a cellular immune response which do not require conformational correct antigen folding [41], it could be hypothesize that this protein, although it had been dialyzed prior to immunization, could not be correctly folded. In addition, SAG1 presented post-translational modifications as glycosylphosphatidylinositol (GPI) additions during its maturation in the parasite [25]. Although in our case it remains to be elucidated whether this post-translational modification occurred during KnS maturation, it has been previously shown that the GPI anchoring motifs does not affect the elicitation of antibody and T-cell responses against this protein [22].

Although our main goal in this study was to evaluate whether plant-derived SAG1 was immunoprotective, we also included a prime-boost protocol with a recombinant SAG1 expressed in bacteria in order to see if we could improve the immunoprotection. Even though the inclusion of a boost increased the protection from 30% to 46% compared to their respective controls and naïve group, the protection observed between these two protocols was not significantly different. The protection observed in the prime-boosted mice is consistent with the significant Th1 humoral and cellular immune responses elicited.

The fact that only with the inclusion of a boost immunization a humoral response was induced, is in agreement with the results observed by Wang et al. [37], who obtained specific antibodies only when plants expressed PyMSP4/5 antigen from *Plasmodium *primed mice where boosted with DNA. It is worth mentioning that the rSAG1 boost immunization induced significant humoral and cellular responses capable of protecting mice against infection, only when mice were previously primed with the SAG1 expressing leaf extracts (SAG1+boost) and not with control extracts (Control+boost). Although similar immune responses were elicited by the Control+boost and SAG1 groups (without the boost), partial protection was only observed in this last group. This protective effect could be attributed to the significant levels of IFN-γ obtained in the SAG1 group. Nevertheless, whether NK or CD8+ T cell cytotoxic activity was induced by the immunization protocols, remains to be elucidated. All these results suggest that immunization with tobacco leaves expressing SAG1 is a reliable system to generate immunity, which could be boosted by heterologous prime-boost inoculation regimens.

Given that *T. gondii *gains entry into the body through the intestinal mucosa, an efficient stimulation of the immune response in this specialized compartment is of great importance for developing a vaccine against this parasite [43]. In this sense, an important purpose of the present work was to test whether leaf-SAG1 oral vaccination could positively influence the outcome of *T. gondii *infection. Oral immunization with SAG1-containing leaves elicited neither evident humoral and cellular immune responses nor protection. Noteworthy, a protocol based on a prime with oral immunizations and a boost with rSAG1 expressed in bacteria injected intradermally elicited an important protection (~50% reduction of the cyst burden when compared to control) that correlated at least with an increased humoral response. It is evident that oral immunization with the plant-derived SAG1 protein prior to the *E. coli*-SAG1 intradermal boost was necessary to elicit significant protection since immunization with control plant extract prior to the boost, nor the boost alone were able to elicit a protective effect. Most of the reports based on oral immunization with plant expressed antigens, elicited a detectable humoral immune response only when an exogenous adjuvant was included in the vaccine formulation and/or high doses of the antigen were used [43-50]. Moreover, some works showed that even using high oral antigen doses in plant extracts with adjuvant, humoral and cellular immune responses were only observed when heterologous prime-boost inoculation regimens were implemented [35,36,40,51]. Taking all these data together, our results are encouraging because a low antigen dose without adjuvant was used. Future studies should consider the evaluation of a wide range of leaf tissue doses using lyophilized leaves [52] and/or the maximization of the efficacy of the antigen presentation by using potent oral adjuvants [53-55].

## Conclusion

We determined that it was feasible to produce vaccine-relevant *T. gondii *SAG1 protein in plants of *Nicotiana *spp. by means of transient expression via recombinant *A. tumefaciens*. We demonstrated that sequence optimization showed a not positive impact on protein yield. The ER targeting improved the native SAG1 protein expression, and it could be used in oral and subcutaneous immunization protocols. SAG1 is one of the most vaccine-relevant *T. gondii *proteins, so it expression in plants could have considerable potential.

## Methods

### Codon usage analysis

The codon usage data of tobacco plants (*Nicotiana tabaccum*) and base composition were obtained from the CUTG (Codon Usage Tabulated from GenBank) website http://www.kazusa.or.jp/codon/. The codon adaptation index (CAI) was used as a parameter to estimate the degree of codon optimization of a whole gene [56]. The value of transgene was calculated with the available software CAI Calculator http://www.cbib.u-bordeaux2.fr/pise/cai.html using tobacco codon usage values.

### Codon modification of the *SAG1 *gene

The design of the modified *SAG1 *gene was based on the data analysis of codon usage. The appropriate codon optimization was performed to make its CAI value and A+T content more similar to the general genes in tobacco plants. The putative transcription termination signals (AAUAAA and its variants) and cryptic splicing sites were also eliminated. Long hairpin loops and A/T strings were avoided to eliminate possible secondary structures that could halt the transcription process and destabilize the mRNA. The sequences and alterations of the *SAG1 *gene are presented in Figure 1. Synthesis of the modified SAG1 ORF (residues 77 to 322, excluding the signal peptide in the N-terminus and the 14 hydrophobic amino acids in the C-terminus) was carried out by the overlapping extension PCR method using the series of overlapping oligos shown in Table 2. The first step consisted of six amplification reactions with pair-wise combinations of the following overlapping sense and antisense primers with their positions in Figure 1 using plasmid pApoSAG1_77-336 _as template [13]. In the following steps, the connected products were amplified by PCR using the previously amplified PCR product as a template. The final full-length products were cloned into the pGemT-Easy vector (Promega). The native SAG1 (residues 77 to 322) was also amplified by PCR from plasmid pApoSAG1_77-336 _and cloned into the pGemT-Easy vector. To ensure that the gene had been amplified without introducing nucleotide errors, both strands were sequenced using primers that were specific for the T7 and SP6 promoters. The synthetic and native genes were appended with the AP24 osmotin signal peptide sequence in the N-terminus for driving the mature protein to the apoplast, and with an endoplasmic reticulum retention signal (TCTGAGAAAGATGAGCTAT) in the C-terminus for the recombinant protein accumulation in the ER. In addition, two restriction sites, *Cla*I and *Eco*RV, were added into the N-terminus and C-terminus, respectively, for the apoplastic versions, and *Cla*I and *Sma*I were added into the N-terminus and C-terminus, respectively, for the ER versions (Figure 1).

### Construction of plant expression vector and Agroinfiltration

The full-length modified and native *SAG1 *genes with different signal peptide sequences were sub-cloned in the expression cassette of intermediate plasmid pBPFΩ7 between the duplicate CaMV 35S promoter (35sCaMV × 2) and the nos terminator (t-NOS). Finally, the entire expression cassette, containing the 35sCaMV × 2, the translational enhancer sequence (Ω), the modified and native sag1 coding sequence and t-NOS, was released and ligated into the binary plant vector pZP200. *Agrobacterium tumefaciens *strain GV3101 (Rif^R ^Gm^R^) was transformed with constructions pnS, poS, pKnS and pKoS (Table 1) using the freeze-thaw method for transformation of *Agrobacterium tumefaciens *[57]. Cultures were grown shaking at 30°C to exponential phase (OD_600 _approx. 0.8) in LB broth containing the appropriate antibiotics. Leaves from 2-4 week-old *Nicotiana tabaccum v Xanthi *plants were infiltrated by vacuum infiltration [58]. Leaves were submerged into the bacterial suspension and subjected to a vacuum of 290 kPa for 5-10 min, with occasional agitation to release trapped air bubbles. The vacuum was released rapidly (approx. 10 kPa). The leaves were placed adaxial side down into Petri dishes containing wet Whatman paper for 4 d (23°C/16 h photoperiod).

### Total soluble protein extraction and Western blot analysis

Tobacco leaves were homogenized in liquid nitrogen with a mortar and a pestle by the addition of an extraction urea buffer (8 M urea, 0.1 M NaH2PO4, 0.01 M Tris-HCl, pH 8.0) in order to solubilize the antigen. The extract was centrifuged at 3000 rpm for 20 min and the supernatant collected and used for expression analyses. For the immunization process, the supernatant was dialyzed against PBS 1X eliminating the urea.

For Western blot analysis, plant extracts were incubated at 100°C for 5 min in loading buffer [59], separated by SDS-PAGE (15% gel) and transferred onto nitrocellulose membranes using an Electro-transfer unit (Bio-Rad). The membranes were sequentially incubated with rabbit polyclonal anti-SAG1 antibody (1:500) and alkaline phosphataseconjugated goat anti-rabbit IgG (1:5,000; Sigma). After washing, the reaction was developed by the addition of nitroblue tetrazolium/5-bromo-4-chloro-3-indolyl phosphate (NBT/BCIP) substrate. Prestained protein (Fermenta) was included in Western blots as molecular weight markers. To estimate the SAG1 accumulation in tobacco leaves *E. coli*-derived SAG1 (rSAG1) of known concentration was also separated in an SDS-PAGE (15% gel) and detected by Western blot (Additional file 1B). The band intensity of purified rSAG1 detected by Western blot was estimated with the "Gel-Pro analyzer" software (Media Cybernetics) and used as a standard to build a calibration curve. The leaf-expressed SAG1 band intensity detected by Western blot was also estimated using the same software and compared with a calibration curve obtained by the band intensity of rSAG1. This strategy allowed us to have an estimation of the concentration of SAG1 expressed in leaf extracts. SAG1 accumulation in tobacco leaves was estimated by Western blot analysis using "Gel-Pro analyzer" as SAG1 μg per percentage of total soluble protein (TSP).

### RT-PCR analysis

Total RNA was isolated from frozen material using Trizol (Gibco) following the manufacturer's instructions. RNA (3-5 μg) was reverse-transcribed using oligo (dT)_18 _primer (Invitrogen) and the SuperScript™ Synthesis System for RT-PCR (Invitrogen). PCR was performed for 30 cycles for both SAG1 and actin using an initial denaturation step at 94°C for 2 min, followed by 30 denaturation (30 s at 94°C), annealing (30 s at 65°C) and extension (1 min at 72°C) cycles. After the last cycle, a final extension was carried out for 10 min at 72°C. PCR products were visualized on 1.2% (w/v) agarose gels using a UV light transilluminator. Oligonucleotides 5'-acgcacagagttgtatgg-3' (SAG1F), 5'-tatcactcgaagcgttacc-3' (SAG1R), 5'-ctggcataatgttgcacttgac-3' (ActF) and 5'-tgcggtggacaatggaaggac-3' (ActR) were used as primers for amplification of the SAG1 and actin coding sequences, respectively. SAG1 transcript levels were normalized with the actin transcripts used as an internal control using "Gel-Pro analyzer" (Media Cybernetics).

### Immunization of mice

Female C3H/HeN (H-2k) and C57BL/6 (H-2b) mice obtained from the Instituto de Investigaciones Biotecnológicas (IIB), Buenos Aires, Argentina, were kept in a pathogen-free environment. The animals were housed according to the NIH Guide for the Care and Use of Laboratory Animals. C3H/HeN mice are resistant to the acute phase of infection by *T. gondii *resulting in brain cyst formation, whereas C57BL/6 mice are sensitive and die 8- 10 days after the oral challenge. Although C3H/HeN presents an intermediate susceptibility to *T. gondii *infection and C57BL/6 is highly sensitive, both are good to produce brain cysts at low parasite doses.

Mice (8 per experimental group) were immunized with four doses of infiltrated leaf extracts, without enrichment or purification, containing the equivalent of approximately 200 ng of SAG1 emulsified in complete (1^st ^dose) or incomplete (2^nd ^- 4^th ^doses) Freund's adjuvant (CFA or IFA) by subcutaneous injection at two-week intervals (SAG1 group). The control group was injected with leaf extracts infiltrated with not transformed *Agrobacterium *plus adjuvant (Control group). Two additional groups were included: one of them was vaccinated as SAG1 group and two weeks later mice were boosted intradermally (ID) with 0.4 μg of rSAG1 expressed in *E. coli *emulsified in IFA (SAG + boost group); the other group was injected as the Control group and boosted ID with 0.4 μg rSAG1 + IFA (Control + boost group).

For oral immunization, three groups of eight C57BL/6 (H-2d) mice were deprived of food and water for 2 h prior to each vaccine dose and then gavaged with 200 μl of plant extract. The SAG1 group received four doses of SAG1-plant extract (200 ng of SAG1 approx.) without adjuvant at one-week interval using an animal feeding needle. The control group received four doses of leaf extracts infiltrated with not transformed *Agrobacterium*. The SAG1 + boost group was immunized as the SAG1 group and one week later, mice were ID boosted with 0.4 μg of rSAG1 expressed in *E. coli *emulsified in IFA. PBS + boost group received four doses of PBS and one week later, mice were ID boosted with 0.4 μg of rSAG1 expressed in *E. coli *emulsified in IFA. In all these experiments a non-immunized group (naïve) was included.

Two weeks after the last immunization, blood was collected from the tail vein and sera were stored at -20°C until analyzed for the presence of specific antibodies. Pre-immune serum samples were used as negative controls.

### Measurement of antibody responses

Antigen-specific antibodies were analyzed by enzyme-linked immunosorbent assay (ELISA) as previously described [13]. Microtiter plates (Immuno Plate Maxisorp; Nunc) were coated with rSAG1 at 5 μg/ml. Goat anti-mouse immunoglobulin G (IgG)-horseradish peroxidase conjugate was used as a secondary antibody (Jackson Immunoresearch Laboratories), or goat anti-mouse IgG1- or IgG2a-horseradish peroxidase conjugates (Pharmingen) were used for isotype analysis. Immune complexes were revealed with OPD and optical density was read at 450 nm with an automatic ELISA reader (Dynatech MR4000). The negative control serum samples included in the assay had values lower than the cut off.

### Measurement of delayed-type hypersensitivity (DTH) response

*T. gondii*-specific delayed-type hypersensitivity (DTH) responses were quantified using a 48-hour *in vivo *hind footpad swelling assay. Two weeks after the last immunizing dose, the right footpad from each mouse was injected with 30 μl of PBS containing 50 μg of TLA. Forty eight hours after challenges, the increase in hind footpad swelling was quantified with a dernier caliper. Results were corrected for prechallenge thickness. The mice selected for DTH responses were not used for any other assay.

### Cytokine analysis

Spleens were aseptically removed from 4 C3H/HeN mice per group two weeks after the last injection. Single-cell preparations were obtained by crushing spleens through stainless steel meshes followed by suspension in erythrocyte lyses buffer (0.15MNH4Cl, 1.0 M KHCO3, 0.1 mM EDTA, pH 7.2). The viability of the cells used in the experiments was always higher than 80%, as measured by trypan blue exclusion (Sigma). The cells were then suspended in RPMI 1640 medium (Life Technologies) supplemented with 10% fetal bovine serum (GIBCO), 2-mercaptoethanol (final concentration, 5 × 10^-5 ^M), penicillin (100 U/ml), and streptomycin (100 μg/ml).

Supernatants from cultured splenocytes (10 × 10^6^/ml) were collected after 72 h of stimulation with rSAG1 (10 μg/ml) and stored at -70°C. The concentrations of secreted IFN-γ was measured by capture ELISA (Pharmingen, BD Biosciences) as specified by the manufacturer. Briefly, microtiter plates (Immuno Plate Maxisorp; Nunc) were coated overnight at 4°C with capturing rat anti-mouse IFN-γ monoclonal antibodies (Pharmingen) diluted in 0.1 M Na2HPO4 (pH 9). Wells were washed thoroughly with PBS-Tween 20 0.05%. Empty binding sites were blocked by 2 h of incubation at 37°C with 1% bovine serum albumin. Supernatants from culture cells were tested in duplicate at 100 μl, and serial dilutions of recombinant murine IFN-γ (Pharmingen) were used at 15-4000 pg/ml for the standard curve. After incubation for 1 h at 37°C, washed plates were incubated with rat biotynilated anti-mouse IFN-γ monoclonal antibodies (Pharmingen) for 1 h. Streptavidin peroxidase conjugate (Sigma) diluted 1:1000 was then added to the washed wells and allowed to react for 30 min. Bound complexes were detected by a solution of 0.15% H_2_O_2_- 0.15% orthophenylene diamine (Sigma) in citrate (0.1 M) - phosphate (0.1 M) buffer (pH 4.5). Absorbance was read at 450 nm in an automatic ELISA reader (Dynatech MR4000). The sensitivity limits for the assays were 20 pg/ml for IFN-γ.

### Challenge infection

C3H/HeN and C57BL/6 mice were orally infected with 20 ME49 tissue cysts (sublethal dose) two weeks after the last immunization. Mice were observed daily for mortality. One month after the challenge, mice were sacrificed and their brains removed. Each brain was homogenized in 2 ml of PBS with a dounce tissue grinder. The mean number of cysts per brain was determined by observation under an optical microscope, by counting four samples of 20 μl aliquots of each homogenized brain.

### Statistical analysis

Statistical analysis was carried out with the Prism 5.0 Software (GraphPad, San Diego, CA) using t' student test or one-way analysis of variance (ANOVA). Values of p < 0.05 were considered significant.

## Authors' contributions

MLB participated in the design of the study, made the codon-optimized SAG1 and SAG1 targeted constructs, carried out the agroinfiltration experiments, analyzed all the recombinant protein expression level data, and helped to carry out the immunology studies. VM planned, supervised and carried out of the immunology studies and helped to draft the manuscript. MK helped to make the SAG1 constructs and did some agroinfiltrations. MCo helped to carry out the western blot and agroinfiltration experiment. MLY helped to carry out the immunology studies. AG helped to supervise and carry out the immunology studies and helped to draft the manuscript. MCl participated in the coordination and design of the study, and drafted the manuscript. All authors read and approved the final manuscript.

## Supplementary Material

Additional file 1 **SAG1 accumulation in tobacco leaves**. (A) Western blot analysis of SAG1 from infiltrated tobacco leaves. Soluble protein samples were electrophoresed on a 15% SDS-PAGE gel and blotted onto a nitrocellulose membrane. The membrane was probed with a rabbit polyclonal anti-SAG1. Coomassie blue was used as a control of the soluble protein samples loaded, which were estimated around 100 μg of total soluble protein by Bradford's assay. The western blot results presented are representative of three independent experiments; (B) Western blot of SAG1 from 300, 200, 100 and 50 μg of PVXS extracts and from *E. coli*-derived SAG1 of known concentration (10, 20 and 30 ng of rSAG1 quantified by Bradford's assay). (C) The SAG1 band intensity expressed from *E. coli *and detected by Western blot was estimated to build a quantization calibration curve with amount standards. The leaf-expressed SAG1 band intensity detected by western blot was estimated using "Gel-Pro analyzer" and compared with the quantification calibration curve; a = p < 0.05: KnS vs. PVXS and AflS, AS vs. KoS, and AnS vs. AoS; b = p < 0.01: PVXS vs. KoS; c = p < 0.001: KnS vs. AoS and KoS, and AnS vs. KoS. (D) Agarose gel of RT-PCR products obtained with primers SAG1F and SAGR and ActF and ActR. The RT- PCR results presented are representative of three independent experiments. GV: pzp200- infiltred leaf extracts; PVXS: pZPVXSAG1-infiltrated leaf extracts; AS: pApoSAG1-infiltrated leaf extracts; AnS and AoS: pAnS and pAoS-infiltrated leaf extracts, respectively; KnS and KoS: pKnS and pKoS-infiltrated leaf extracts, respectively. Arrows indicate the bands of 35 kDa and 19 kDa detected with the anti-SAG1 antibody in the plant extract expressing SAG1. M: prestained protein molecular marker. Click here for file

Additional file 2 **Protection assay after challenge with *T. gondii *cysts in orally immunized C57BL/6 (H-2d) mice**. Eight- to ten-week-old mice (8/group) were immunized on days 0, 7, 14 and 21 by oral vaccination. Two weeks after the last boost, mice were challenged by gavage with 20 cysts of the Me49 strain (LD50). Thirty days later, the number of brain cysts in mice was determined. Control: mice orally vaccinated with pzp200-infiltrated leaf extracts, PBS+Boost: mice orally inoculated with 3 doses of PBS and a final intradermal boost with rSAG1. Click here for file

## References

[B1] Floss DM Falkenburg D Conrad U Production of vaccines and therapeutic antibodies for veterinary applications in transgenic plants: an overview Transgenic Res 2007 16 315 332 10.1007/s11248-007-9095-x 17436059 PMC7089296

[B2] Yusibov V Rabindran S Recent progress in the development of plant derived vaccines Expert Rev Vaccines 2008 7 1173 1183 10.1586/14760584.7.8.1173 18844592

[B3] Doran PM Foreign protein degradation and instability in plants and plant tissue cultures Trends Biotechnol 2006 24 426 432 10.1016/j.tibtech.2006.06.012 16843560

[B4] Streatfield SJ Approaches to achieve high-level heterologous protein production in plants Plant Biotechnol J 2007 5 2 15 10.1111/j.1467-7652.2006.00216.x 17207252

[B5] Tenter AM Heckeroth AR Weiss LM *Toxoplasma gondii*: from animals to humans Int J Parasitol 2000 30 1217 1258 10.1016/S0020-7519(00)00124-7 11113252 PMC3109627

[B6] Luft BJ Remington JS Toxoplasmic encephalitis in AIDS Clin Infect Dis 1992 15 211 222 1520757 10.1093/clinids/15.2.211

[B7] Rorman E Zamir CS Rilkis I Ben-David H Congenital toxoplasmosis--prenatal aspects of *Toxoplasma gondii *infection Reprod Toxicol 2006 21 458 472 10.1016/j.reprotox.2005.10.006 16311017

[B8] Dubey JP Schlafer DH Urban JF Jr Lindsay DS Lesions in fetal pigs with transplacentally-induced toxoplasmosis Vet Pathol 1990 27 411 418 10.1177/030098589902700605 2278129

[B9] Buxton D Protozoan infections (*Toxoplasma gondii*, *Neospora caninum *and *Sarcocystis spp*.) in sheep and goats: recent advances Vet Res 1998 29 289 310 9689743

[B10] Wyss R Sager H Müller N Inderbitzin F König M Audigé L Gottstein B The occurrence of *Toxoplasma gondii *and *Neospora caninum *as regards meat hygiene Schweiz Arch Tierheilkd 2000 142 95 108 10748708

[B11] Qu D Wang S Cai W Du A Protective effect of a DNA vaccine delivered in attenuated *Salmonella typhimurium *against *Toxoplasma gondii *infection in mice Vaccine 2008 26 4541 4548 10.1016/j.vaccine.2008.06.030 18590785

[B12] Bhopale GM Development of a vaccine for toxoplasmosis: current status Microbes Infect 2003 5 457 462 10.1016/S1286-4579(03)00048-0 12738002

[B13] Clemente M Curilovic R Sassone A Zelada A Angel SO Mentaberry AN Production of the main surface antigen of *Toxoplasmagondii *in tobacco leaves and analysis of its antigenicity and immunogenicity Mol Biotechnol 2005 30 41 50 10.1385/MB:30:1:041 15805575

[B14] Ferraro G Becher ML Angel SO Zelada A Mentaberry AN Clemente M Efficient expression of a *Toxoplasma gondii *dense granule Gra4 antigen in tobacco leaves Exp Parasitol 2008 120 118 122 10.1016/j.exppara.2008.06.002 18588877

[B15] Letscher Bru V Villard O Risse B Zauke M Klein JP Kien TT Protective effect of vaccination with a combination of recombinant surface antigen 1 and interleukin 12 against toxoplasmosis in mice Infect Immun 1998 66 4503 4506 9712808 10.1128/iai.66.9.4503-4506.1998 PMC108546

[B16] Haumont M Delhaye L Garcia L Jurado M Mazza P Daminet V Verlant V Bollen A Biemans R Jacquet A Protective immunity against congenital toxoplasmosis with recombinant SAG1 protein in a Guinea pig model Infect Immun 2000 68 4948 4953 10.1128/IAI.68.9.4948-4953.2000 10948109 PMC101707

[B17] Nielsen HV Lauemoller SL Christiansen L Buus S Fomsgaard A Petersen E Complete protection against lethal *Toxoplasma gondii *infection in mice immunized with a plasmid encoding the SAG1 gene Infect Immun 1999 67 6358 6363 10569750 10.1128/iai.67.12.6358-6363.1999 PMC97042

[B18] Angus CW Klivington-Evans D Dubey JP Kovacs JA Immunization with a DNA plasmid encoding the SAG1 (P30) protein of *Toxoplasma gondii *is immunogenic and protective in rodents J Infect Dis 2000 181 317 324 10.1086/315186 10608781

[B19] Petersen E Henrik VN Christiansen L Spenter J Immunization with *E. coli *produced recombinant *T. gondii *SAG1 with alum as adjuvant protects mice against lethal infection with *Toxoplasma gondii* Vaccine 1998 16 1283 89 10.1016/S0264-410X(98)00039-5 9682392

[B20] Chen G Chen H Guo H Zheng H Protective effect of DNA-mediated immunization with a combination of SAG1 and IL-2 gene adjuvant against infection of *Toxoplasma gondii *in mice Chin Med J (Engl) 2002 115 1448 1452 12490084

[B21] Couper KN Nielsen HV Petersen E Roberts F Roberts CW Alexander J DNA vaccination with the immunodominant tachyzoite surface antigen (SAG-1) protects against adult acquired *Toxoplasma gondii *infection but does not prevent maternofetal transmission Vaccine 2003 21 2813 2820 10.1016/S0264-410X(03)00163-4 12798622

[B22] Caetano BC Bruña-Romero O Fux B Mendes EA Penido ML Gazzinelli RT Vaccination with replication-deficient recombinant adenoviruses encoding the main surface antigens of *Toxoplasma gondii *induces immune response and protection against infection in mice Hum Gene Ther 2006 17 415 426 10.1089/hum.2006.17.415 16610929

[B23] Zhou H Gu Q Zhao Q Zhang J Cong H Li Y Shenyi H *Toxoplasma gondii*: expression and characterization of a recombinant protein containing SAG1 and GRA2 in *Pichia pastoris* Parasitol Res 2007 100 829 835 10.1007/s00436-006-0341-6 17058109

[B24] Kasper LH Khan IA Ely KH Buelow R Boothroyd JC Antigen-specific (p30) mouse CD8+ T cells are cytotoxic against *Toxoplasma gondii*-infected peritoneal macrophages J Immunol 1992 148 1493 1498 1538132

[B25] Nagel SD Boothroyd JC The major surface antigen, P30, of *Toxoplasma gondii *is anchored by a glycolipid J Biol Chem 1989 264 5569 5574 2925621

[B26] Mallory AC Parks G Endres MW Baulcombe D Bowman LH Pruss GJ Vance VB The amplicon-plus system for high-level expression of transgenes in plants Nat Biotechnol 2002 20 622 625 10.1038/nbt0602-622 12042869

[B27] Voinnet O Rivas S Mestre P Baulcombe D An enhanced transient expression system in plants based on suppression of gene silencing by the p19 protein of tomato bushy stunt virus Plant J 2003 33 949 956 10.1046/j.1365-313X.2003.01676.x 12609035

[B28] Bayne EH Rakitina DV Morozov SY Baulcombe DC Cell-to-cell movement of potato potexvirus X is dependent on suppression of RNA silencing Plant J 2005 44 471 482 10.1111/j.1365-313X.2005.02539.x 16236156

[B29] Graille M Stura EA Bossus M Muller BH Letourneur O Battail-Poirot N Sibaï G Gauthier M Rolland D Le Du MH Ducancel F Crystal structure of the complex between the monomeric form of *Toxoplasma gondii *surface antigen 1 (SAG1) and a monoclonal antibody that mimics the human immune response J Mol Biol 2005 354 447 458 10.1016/j.jmb.2005.09.028 16242717

[B30] De Rocher EJ Vargo-Gogola TC Diehn SH Green PJ Direct evidence for rapid degradation of *Bacillus thuringiensis *toxin mRNA as a cause of poor expression in plants Plant Physiol 1998 117 1445 1461 10.1104/pp.117.4.1445 9701600 PMC34908

[B31] Gutiérrez RA MacIntosh GC Green PJ Current perspectives on mRNA stability in plants: multiple levels and mechanisms of control Trends plant Sci 1999 4 429 438 10.1016/S1360-1385(99)01484-3 10529824

[B32] Hollams EM Giles KM Thomson AM Leedman PJ MRNA stability and the control of gene expression: implications for human disease Neurochem Res 2002 27 957 980 10.1023/A:1020992418511 12462398

[B33] Ahmed R Duncan RF Translational regulation of Hsp90v RNA. AUG-proximal 5'-untranslated region elements essential for preferential heat shock translation J Biol Chem 2004 279 49919 49930 10.1074/jbc.M404681200 15347681

[B34] Geyer BC Fletcher SP Griffin TA Lopker MJ Soreq H Mor TS Translational control of recombinant human acetylcholinesterase accumulation in plants BMC Biotechnol 2007 30 7 27 10.1186/1472-6750-7-27 PMC1913049 17537261

[B35] Suo G Chen B Zhang J Duan Z He Z Yao W Yue C Dai J Effects of codon modification on human BMP2 gene expression in tobacco plants Plant Cell Rep 2006 25 689 697 10.1007/s00299-006-0133-6 16491379

[B36] Maclean J Koekemoer M Olivier AJ Stewart D Hitzeroth II Rademacher T Fischer R Williamson AL Rybicki EP Optimization of human papillomavirus type 16 (HPV-16) L1 expression in plants: comparison of the suitability of different HPV-16 L1 gene J Gen Virol 2007 88 1460 1469 10.1099/vir.0.82718-0 17412974

[B37] Wang L Webster DE Campbell AE Dry IB Wesselingh SL Coppel RL Immunogenicity of *Plasmodium yoelii *merozoite surface protein 4/5 produced in transgenic plants Int J Parasitol 2008 38 103 110 10.1016/j.ijpara.2007.06.005 17681344

[B38] Webster DE Wang L Mulcair M Ma C Santi L Mason HS Wesselingh SL Coppel RL Production and characterization of an orally immunogenic *Plasmodium *antigen in plants using a virus-based expression system Plant Biotechnol J 2009 7 846 855 10.1111/j.1467-7652.2009.00447.x 19781007

[B39] Wandelt CI Khan MR Craig S Schroeder HE Spencer D Higgins TJ Vicilin with carboxy-terminal KDEL is retained in the endoplasmic reticulum and accumulates to high levels in the leaves of transgenic plants Plant J 1992 2 181 192 1302048 10.1046/j.1365-313x.1992.t01-41-00999.x

[B40] Yoshida K Matsui T Shinmyo A The plant vesicular transport engineering for production of useful recombinant proteins J Mol Catalysis B: Enzymatic 2004 28 167 171 10.1016/j.molcatb.2004.01.017

[B41] Sher A Collazzo C Scanga C Jankovic D Yap G Aliberti J Induction and regulation of IL-12-dependent host resistance to *Toxoplasma gondii* Immunol Res 2003 27 521 528 10.1385/IR:27:2-3:521 12857995

[B42] Harning D Spenter J Metsis A Vuust J Petersen E Recombinant *Toxoplasma gondii *surface Antigen 1 (P30) expressed in *Escherichia coli *is recognized by human Toxoplasma-specific immunoglobulin M (IgM) and IgG antibodies Clin Diagn Lab Immunol 1996 3 355 357 8705683 10.1128/cdli.3.3.355-357.1996 PMC170346

[B43] Bout DT Mevelec MN Velge-Roussel F Dimier-Poisson I Lebrun M Prospects for a human *Toxoplasma *vaccine Curr Drug Targets Immune Endocr Metabol Disord 2002 2 227 234 10.2174/1568008023340488 12476487

[B44] Kong Q Richter L Yang YF Arntzen CJ Mason HS Thanavala Y Oral immunization with hepatitis B surface antigen expressed in transgenic plants Proc Natl Acad Sci USA 2001 98 11539 11544 10.1073/pnas.191617598 11553782 PMC58765

[B45] Franconi R Di Bonito P Dibello F Accardi L Muller A Cirilli A Simeone P Donà MG Venuti A Giorgi C Plant-derived human papillomavirus 16 E7 oncoprotein induces immune response and specific tumor protection Cancer Res 2002 62 3654 3658 12097270

[B46] Guerrero-Andrade O Loza-Rubio E Olivera-Flores T Fehervari-Bone T Gomez-Lim MA Expression of the Newcastle disease virus fusion protein in transgenic maize and immunological studies Transgenic Res 2006 15 455 463 10.1007/s11248-006-0017-0 16906446

[B47] Gómez E Chimeno Zoth S Carrillo E Estela Roux M Berinstein Mucosal immunity induced by orally administered transgenic plants Immunobiology 2008 213 671 675 10.1016/j.imbio.2008.02.002 18950595

[B48] Santi L Batchelor L Huang Z Hjelm B Kilbourne J Arntzen CJ Chen Q Mason HS An efficient plant viral expression system generating orally immunogenic *Norwalk virus*-like particles Vaccine 2008 26 1846 1854 10.1016/j.vaccine.2008.01.053 18325641 PMC2744496

[B49] Zhang X Yuan Z Duan Q Zhu H Yu H Wang Q Mucosal immunity in mice induced by orally administered transgenic rice Vaccine 2009 27 1596 1600 10.1016/j.vaccine.2008.12.042 19146896

[B50] Matsumoto Y Suzuki S Nozoye T Yamakawa T Takashima Y Arakawa T Tsuji N Takaiwa F Hayashi Y Oral immunogenicity and protective efficacy in mice of transgenic rice plants producing a vaccine candidate antigen (As16) of *Ascaris suum *fused with cholera toxin Bsubunit Transgenic Res 2009 18 185 192 10.1007/s11248-008-9205-4 18763047

[B51] Mayers A Chakauya E Shephard E Tanzer FL Maclean J Lynch A Williamson AL Rybicki EP Expression of HIV-1 antigens in plants as potential subunit vaccines BMC Biotechnology 2008 53 1 15 10.1186/1472-6750-8-53 PMC2443125 18573204

[B52] Kostrzak A Cervantes Gonzalez M Guetard D Nagaraju DB Wain-Hobson S Tepfer D Pniewski T Sala M Oral administration of low doses of plant-based HBsAg induced antigen-specific IgAs and IgGs in mice, without increasing levels of regulatory T cells Vaccine 2009 27 4798 4807 10.1016/j.vaccine.2009.05.092 19539581

[B53] Quintana FJ Cohen RI Heat Shock Proteins as Endogenous Adjuvants in Sterile and Septic Inflammation J Immunol 2005 175 2777 2782 16116161 10.4049/jimmunol.175.5.2777

[B54] Dong JL Liang BG Jin YS Zhang WJ Wang T Oral immunization with pBpoSP6-transgenic alfalfa protects mice against rotavirus infection Virology 2005 339 153 163 10.1016/j.virol.2005.06.004 15992851

[B55] Zimmermann S Dalpke A Heeg K CpG oligonucleotides as adjuvant in therapeutic vaccines against parasitic infections Int J Med Microbiol 2008 298 39 44 10.1016/j.ijmm.2007.07.011 17716944

[B56] Sharp PM Li WH The codon Adaptation Index-a measure of directional synonymous codon usage bias, and its potential applications Nucleic Acids Res 1987 15 1281 1295 10.1093/nar/15.3.1281 3547335 PMC340524

[B57] Höfgen R Willmitzer L Storage of competent cells for *Agrobacterium *transformation Nucleic Acids Res 1988 16 9877 10.1093/nar/16.20.9877 3186459 PMC338805

[B58] Kapila J De Rycke R van Montagu M Angenon G An *Agrobacterium*-mediated transient gene expression system in intact leaves Plant Sci 1997 122 101 108 10.1016/S0168-9452(96)04541-4

[B59] Sambrook J Fritsch EF Maniatis T Molecular cloning: a laboratory manual 1989 Cold Spring Harbor Press

