# Supplementary material for: Effect of codon optimization and subcellular targeting on Toxoplasma gondii antigen SAG1 expression in tobacco leaves to use in subcutaneous and oral immunization in mice
Source: BMC Biotechnol. 2010 Jul 15;10:52. doi: 10.1186/1472-6750-10-52 (PMC2920232; doi:10.1186/1472-6750-10-52)
Supplement: Additional file 1 — SAG1 accumulation in tobacco leaves. (A) Western blot analysis of SAG1 from infiltrated tobacco leaves. Soluble protein samples were electrophoresed on a 15% SDS-PAGE gel and blotted onto a nitrocellulose membrane. The membrane was probed with a rabbit polyclonal anti-SAG1. Coomassie blue was used as a control of the soluble protein samples loaded, which were estimated around 100 μg of total soluble protein by Bradford's assay. The western blot results presented are representative of three independent experiments; (B) Western blot of SAG1 from 300, 200, 100 and 50 μg of PVXS extracts and from E. coli-derived SAG1 of known concentration (10, 20 and 30 ng of rSAG1 quantified by Bradford's assay). (C) The SAG1 band intensity expressed from E. coli and detected by Western blot was estimated to build a quantization calibration curve with amount standards. The leaf-expressed SAG1 band intensity detected by western blot was estimated using "Gel-Pro analyzer" and compared with the quantification calibration curve; a = p < 0.05: KnS vs. PVXS and AflS, AS vs. KoS, and AnS vs. AoS; b = p < 0.01: PVXS vs. KoS; c = p < 0.001: KnS vs. AoS and KoS, and AnS vs. KoS. (D) Agarose gel of RT-PCR products obtained with primers SAG1F and SAGR and ActF and ActR. The RT- PCR results presented are representative of three independent experiments. GV: pzp200- infiltred leaf extracts; PVXS: pZPVXSAG1-infiltrated leaf extracts; AS: pApoSAG1-infiltrated leaf extracts; AnS and AoS: pAnS and pAoS-infiltrated leaf extracts, respectively; KnS and KoS: pKnS and pKoS-infiltrated leaf extracts, respectively. Arrows indicate the bands of 35 kDa and 19 kDa detected with the anti-SAG1 antibody in the plant extract expressing SAG1. M: prestained protein molecular marker. [file 1472-6750-10-52-S1.PPT]

## Slide 1
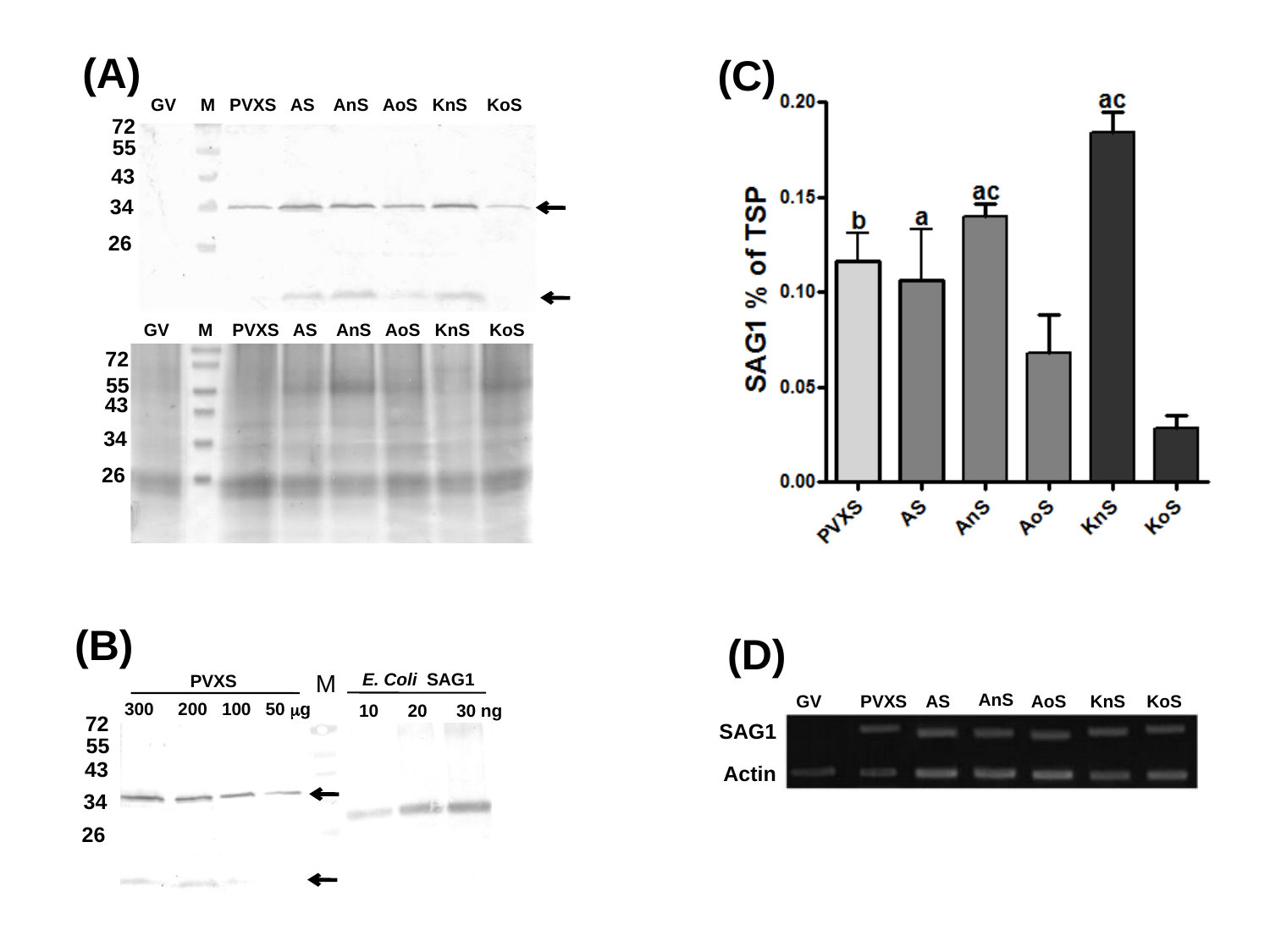

(A)
 GV M PVXS AS AnS AoS KnS KoS
 GV M PVXS AS AnS AoS KnS KoS
(C)
72
55
43
34
26
72
55
43
34
26
(B)
E. Coli SAG1
PVXS
300 200 100 50 μg
 10 20 30 ng
M
(D)
 AnS
GV PVXS AS
KoS
 AoS
 KnS
SAG1
Actin
72
55
43
34
26
