# Supplementary figures and images for: Effect of codon optimization and subcellular targeting on Toxoplasma gondii antigen SAG1 expression in tobacco leaves to use in subcutaneous and oral immunization in mice
Source: BMC Biotechnol. 2010 Jul 15;10:52. doi: 10.1186/1472-6750-10-52 (PMC2920232; doi:10.1186/1472-6750-10-52)

## Slide 1
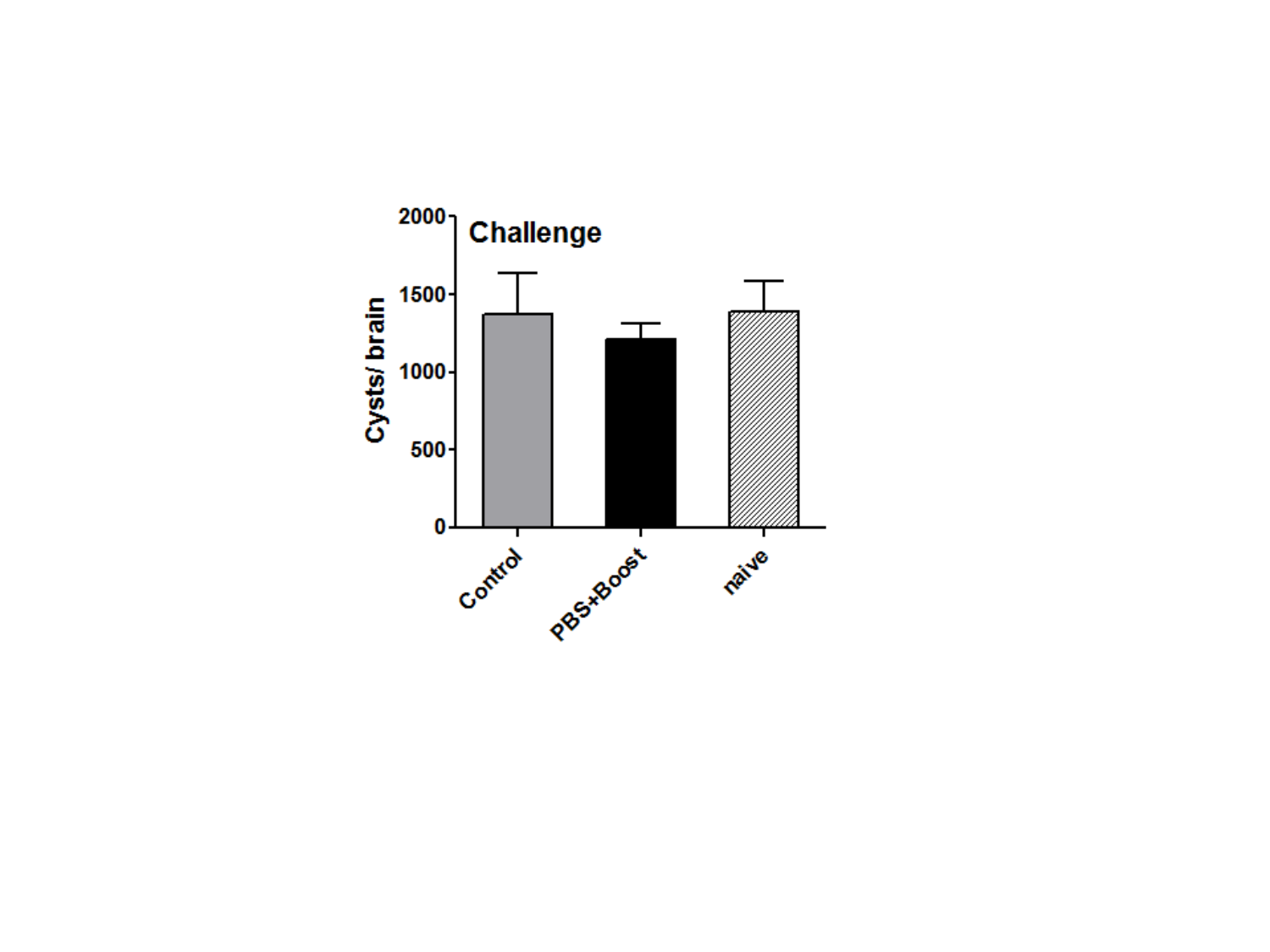

Supplement: Additional file 2 — Protection assay after challenge with T. gondii cysts in orally immunized C57BL/6 (H-2d) mice. Eight- to ten-week-old mice (8/group) were immunized on days 0, 7, 14 and 21 by oral vaccination. Two weeks after the last boost, mice were challenged by gavage with 20 cysts of the Me49 strain (LD50). Thirty days later, the number of brain cysts in mice was determined. Control: mice orally vaccinated with pzp200-infiltrated leaf extracts, PBS+Boost: mice orally inoculated with 3 doses of PBS and a final intradermal boost with rSAG1. [file 1472-6750-10-52-S2.PPT]
